# A Fully Automated Analytic System for Measuring Endolymphatic Hydrops Ratios in Patients With Ménière Disease via Magnetic Resonance Imaging: Deep Learning Model Development Study

**DOI:** 10.2196/29678

**Published:** 2021-09-21

**Authors:** Chae Jung Park, Young Sang Cho, Myung Jin Chung, Yi-Kyung Kim, Hyung-Jin Kim, Kyunga Kim, Jae-Wook Ko, Won-Ho Chung, Baek Hwan Cho

**Affiliations:** 1 Department of Digital Health Samsung Advanced Institute for Health Sciences & Technology Sungkyunkwan University Seoul Republic of Korea; 2 Department of Otorhinolaryngology-Head and Neck Surgery Samsung Medical Center Sungkyunkwan University School of Medicine Seoul Republic of Korea; 3 Department of Radiology Samsung Medical Center Sungkyunkwan University School of Medicine Seoul Republic of Korea; 4 Biomedical Statistics Center, Data Science Research Institute Research Institute for Future Medicine Samsung Medical Center Seoul Republic of Korea; 5 Department of Clinical Pharmacology and Therapeutics Samsung Medical Center Seoul Republic of Korea; 6 Department of Medical Device Management and Research Samsung Advanced Institute for Health Sciences & Technology Sungkyunkwan University Seoul Republic of Korea

**Keywords:** deep learning, magnetic resonance imaging, medical image segmentation, Ménière disease, inner ear, endolymphatic hydrops, artificial intelligence, machine learning, multi-class segmentation, convolutional neural network, end-to-end system, clinician support, clinical decision support system, image selection, clinical usability, automation

## Abstract

**Background:**

Recently, the analysis of endolymphatic hydropses (EHs) via inner ear magnetic resonance imaging (MRI) for patients with Ménière disease has been attempted in various studies. In addition, artificial intelligence has rapidly been incorporated into the medical field. In our previous studies, an automated algorithm for EH analysis was developed by using a convolutional neural network. However, several limitations existed, and further studies were conducted to compensate for these limitations.

**Objective:**

The aim of this study is to develop a fully automated analytic system for measuring EH ratios that enhances EH analysis accuracy and clinical usability when studying Ménière disease via MRI.

**Methods:**

We proposed the 3into3Inception and 3intoUNet networks. Their network architectures were based on those of the Inception-v3 and U-Net networks, respectively. The developed networks were trained for inner ear segmentation by using the magnetic resonance images of 124 people and were embedded in a new, automated EH analysis system—inner-ear hydrops estimation via artificial intelligence (INHEARIT)-version 2 (INHEARIT-v2). After fivefold cross-validation, an additional test was performed by using 60 new, unseen magnetic resonance images to evaluate the performance of our system. The INHEARIT-v2 system has a new function that automatically selects representative images from a full MRI stack.

**Results:**

The average segmentation performance of the fivefold cross-validation was measured via the intersection of union method, resulting in performance values of 0.743 (SD 0.030) for the 3into3Inception network and 0.811 (SD 0.032) for the 3intoUNet network. The representative magnetic resonance slices (ie, from a data set of unseen magnetic resonance images) that were automatically selected by the INHEARIT-v2 system only differed from a maximum of 2 expert-selected slices. After comparing the ratios calculated by experienced physicians and those calculated by the INHEARIT-v2 system, we found that the average intraclass correlation coefficient for all cases was 0.941; the average intraclass correlation coefficient of the vestibules was 0.968, and that of the cochleae was 0.914. The time required for the fully automated system to accurately analyze EH ratios based on a patient's MRI stack was approximately 3.5 seconds.

**Conclusions:**

In this study, a fully automated full-stack magnetic resonance analysis system for measuring EH ratios was developed (named INHEARIT-v2), and the results showed that there was a high correlation between the expert-calculated EH ratio values and those calculated by the INHEARIT-v2 system. The system is an upgraded version of the INHEARIT system; it has higher segmentation performance and automatically selects representative images from an MRI stack. The new model can help clinicians by providing objective analysis results and reducing the workload for interpreting magnetic resonance images.

## Introduction

Although many medical fields have been developed over the past few decades, medical imaging techniques, such as computed tomography and magnetic resonance imaging (MRI), have advanced greatly [1]. Experienced radiologists and physicians typically interpret such images in the clinical field. However, in recent years, due to the remarkable development of machine learning, the situation is changing [2]. Artificial intelligence, including machine learning, is widely used in various fields of medical science, and image analysis via a convolutional neural network is developing rapidly [3].

Ménière disease is a multifactorial disorder with typical symptoms, such as recurrent vertigo attacks, fluctuating hearing loss, tinnitus, and sensations of ear fullness. The prevalence of Ménière disease varies by region and study, but the estimated prevalence ranges from 30 to 150 patients per 100,000 people [4]. In particular, it is known that this prevalence is higher within White and female populations and increases with age [5]. Endolymphatic hydrops (EH) is a histologic hallmark of Ménière disease in which the endolymphatic spaces in the cochlea and the inner ear vestibule are distended [6]. According to current diagnostic criteria, pure tone audiometry is the only objective test for the diagnosis of definite or probable Ménière disease. Further, electrocochleography is a common test for estimating EH ratios [7]. However, electrocochleography is used only as a reference examination for diagnosing Ménière disease because it does not directly show the endolymphatic space. Similarly, Ménière disease is challenging to diagnose objectively, and efforts have been made in recent years to directly measure EH ratios by using MRI [8-10].

A protocol for image-based EH analysis was suggested in a previous study [11]; it required specific image viewer software to generate a hybrid image of signals (ie, a hybrid of the reversed image of the positive endolymph signal and native image of the positive perilymph signal [HYDROPS] or a HYDROPS image multiplied by T_2_-weighted magnetic resonance cisternography [HYDROPS-Mi2]). This process involves the manual contouring of inner ear organs for boundary segmentation, which is mostly performed by medical experts.

**Figure 1:**
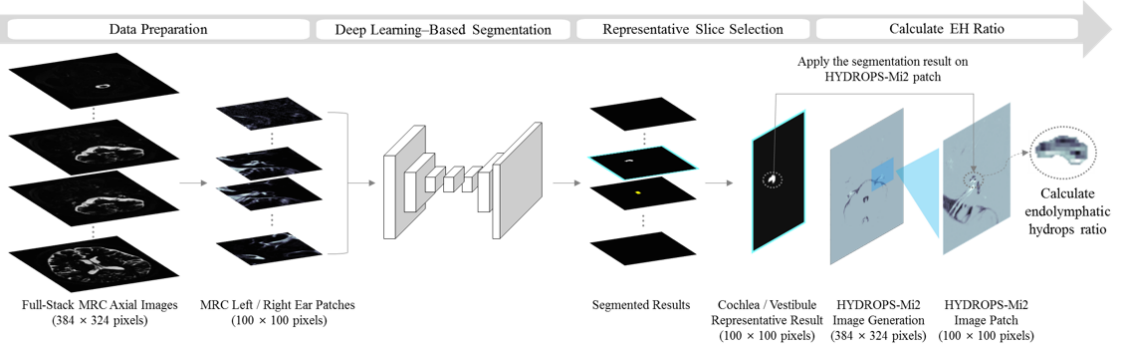
The proposed inner-ear hydrops estimation via artificial intelligence-version 2 (INHEARIT-v2) framework. EH: endolymphatic hydrops; HYDROPS-Mi2: hybrid of the reversed image of the positive endolymph signal and native image of the positive perilymph signal image multiplied by T_2_-weighted magnetic resonance cisternography; MRC: magnetic resonance cisternography.

The need for automated analyses has emerged because conventional quantitative analyses require more time and effort than typical image interpretation processes. The automatic measurement of EH ratios via MRI was proposed based on the deep learning approach in our previous study [12]. Our research showed that the convolutional neural network–based deep learning model—the inner-ear hydrops estimation via artificial intelligence (INHEARIT) system—could efficiently segment cochleae and vestibules in magnetic resonance images and calculate the EH ratios of the segmented regions [12]. However, our study had a few limitations. First, full-stack image validation was not conducted in our previous study. Thus, medical experts were needed to manually select representative image slices of cochleae and vestibules from the full magnetic resonance image stack and load them into the system, and this human user process was time consuming. Second, validation with an isolated data set was not performed, which made it difficult to verify the robustness of the system. Lastly, various deep learning models were not used, except for the Visual Geometry Group (VGG) network architecture—a VGG-19–based network [13]. To compensate for these limitations, we developed a fully automated analytic system for calculating EH ratios by using deep learning and MRI—the INHEARIT-version 2 (INHEARIT-v2) system. The entire framework for this analytic system is depicted in Figure 1.

## Methods

### Study Participants

Two data sets were used in this study—one for the deep learning–based training models and another for validation. For the training models, the magnetic resonance images of 124 patients (males: n=57; females: n=67; age: mean 49.3 years, SD 16.02 years; range 17-76 years) who participated in our previous INHEARIT study were used [12]. For the additional test, 60 new participants were recruited from Samsung Medical Center from February 2018 to September 2019. Written informed consent was obtained from all participants. This study was approved by the Institutional Review Board of Samsung Medical Center (approval number: 2020-06-046).

### Data Set for Analysis

We scanned all patients’ inner ears with a 3.0-T MRI device (MAGNETOM Skyra; Siemens Medical Solutions). Gadobutrol (gadolinium-DO3A-butriol; Gadovist 1.0), which was used as the contrast agent, was intravenously administered to patients before MRI. Different magnetic resonance scans were made by using heavily T_2_-weighted magnetic resonance cisternography (MRC) to distinguish the outlines of organs. Positive perilymph images (PPIs) and positive endolymph images (PEIs) were used to evaluate EHs. The HYDROPS images were obtained by subtracting the number of PEIs from the number of PPIs. MRC images, PPIs, and PEIs had identical fields of view, matrix sizes, and slice thicknesses [8,14].

### Data Annotation by Physicians

One neuroradiologist and one neuro-otologist independently evaluated the MRC images. Representative slices of 4 classes—the left cochlea and vestibule and the right cochlea and vestibule—were selected, and their contours were annotated manually. The main slices of a cochlea or vestibule and their regions of interest (ROIs) were chosen based on the following criteria. For the cochlear ROI, among all MRI slices in which the basal, middle, and apical views of organs were visible, the slice with the largest modiolus height was chosen. For the vestibular ROI, the lowest slice, in which the lateral semicircular canal ring was obvious in images that were rotated by more than 240°, was chosen, and images of the ampulla were excluded [12]. Each physician drew contours of the ROIs on MRC images, and the contours were compared to determine a point of agreement.

### Deep Learning Training Models for Inner Ear Segmentation

To segment the ROIs, 2 deep learning models were developed based on the architectures of the Inception-v3 [15] and U-Net [16] networks. The models were named 3into3Inception and 3intoUNet. For segmentation via the Inception-v3 network, deconvolutional layers were added after the conventional feature extraction of convolutional layers, and skip connections were included between convolutional and corresponding deconvolutional layers, as we did in our previous study [12]. The purpose of the U-Net network is to conduct segmentation; this network consists of feature contraction and feature expansion parts that function as encoders and decoders, respectively. In this network, a portion of contracted information is copied and concatenated to the corresponding expansion part, thereby reducing the amount of information that is lost during the segmentation process.

The physician-selected main slice image and the previous and next images were loaded simultaneously from full-stack MRC images for model training. The cross-sectional area of target inner ear structures is relatively small in a whole-brain image. Since this can cause a class imbalance problem, image patches of 100×100 pixels were acquired from each left and right reference point. This process is shown with the 3intoUNet network in Multimedia Appendix 1; 3 sequential MRC image patches were independently fed into each of the networks, and features were summated before the addition of deconvolutional layers. The green dotted boxes represent parts of the network that perform the same function (ie, the contraction of features in each input image). This sequential 3-input approach has been shown to yield high performance in terms of medical image segmentation [12]. The implementation and analyses of these models were performed via the Python 3.5 (Python Software Foundation) environment. The NumPy library was used for arithmetic calculation, the sklearn and ImageIO libraries were used for image preprocessing, and the TensorFlow library was used for model training.

The developed models were trained with a selectively annotated data set of 124 subjects. Afterward, the models were fine-tuned with both the selectively annotated and fully annotated data sets, per the optimum curriculum learning strategy for the segmentation of weakly annotated data [12]. Although we analyzed 4 target organs (the left cochlea, left vestibule, right cochlea, and right vestibule), the number of representative image slices per subject varied between 2 and 4 because a cochlea and a vestibule from each side could have been in the same image slice depending on the anatomical structure of the person. First, we trained a model with the selectively annotated data set and conducted moderate augmentation (1584 times). Afterward, we fine-tuned the model with both the fully annotated and selectively annotated data sets and conducted high augmentation (14,544 times). Therefore, the original 262 slices from the selectively annotated data set were augmented to 412,008 slices, and the initial 372 slices from the fully annotated and selectively annotated data sets were augmented to 5,410,368 slices for training. For augmentation, image patches were randomly flipped along the horizontal direction, cropped via random shifting (ie, from a reference point), and had their pixel intensity changed. There was a wide range of variation between the augmented images and the original image (eg, differences in intensity, shifted cropping areas, etc), and the physicians agreed to use the augmented images as training inputs.

Fivefold cross-validation was conducted, and segmentation performance was evaluated by measuring the intersection over union (IoU) between the ground truth areas (clinician-annotated region) and prediction areas (model-based, automatically determined region). The IoU was calculated as follows:

IoU = area of overlap/area of union = *A_overlap_*/(*A_GT_* + *A_pred_* − *A_overlap_*) **(1)**

In equation 1, *A_GT_* is the ground truth area, *A_pred_* is the prediction area, and *A_overlap_* is the intersection between *A_GT_* and *A_pred_*. The model was trained on graphical processing units (NVIDIA GTX 1080Ti; Nvidia Corporation). The parameters were determined via grid searching and optimized to a learning rate of 1e^−5^ with the Adam optimizer [17]; a dropout rate [18] of 0.4 and batch size of 4 were used. Batch normalization and mean subtractions were performed to prevent internal covariate shifts.

### Full-Stack Image Segmentation of an Additional Test Data Set

Once model training was completed, the model was tested with an additional data set of unseen magnetic resonance images. As shown in Figure 1, the full-stack MRC images of a patient were fed into a deep learning network. Slice indices that represented cochleae and vestibules were selected from a stack of segmentation results. The selected results were applied to HYDROPS-Mi2 patches as masks, and EH ratios were calculated for segmented regions.

**Figure 2:**
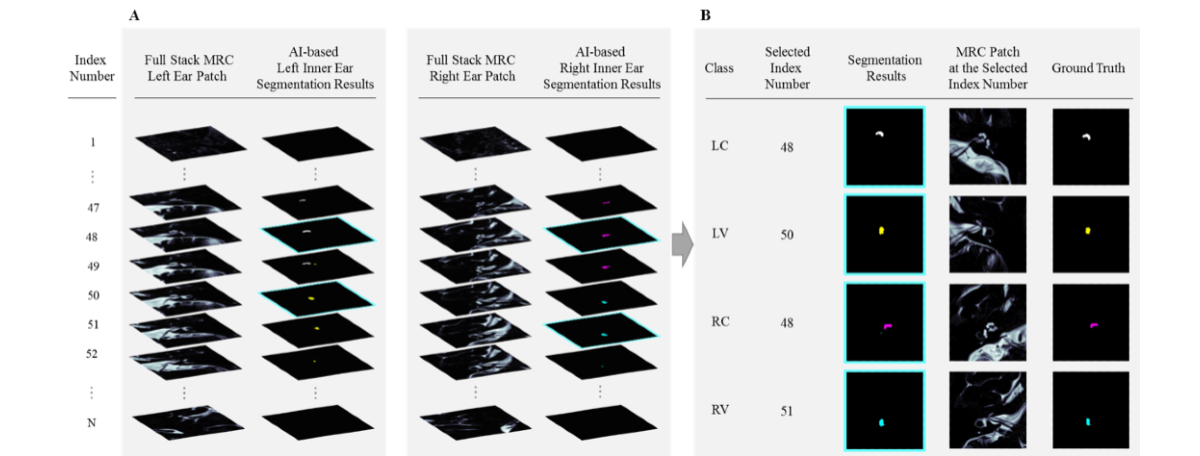
The full-stack magnetic resonance image segmentation analysis process for the test data set. A: A full stack of MRC images and their segmentation results. B: Representative slices and the segmentation results of each class. AI: artificial intelligence; MRC: magnetic resonance cisternography.

Figure 2 shows the process of conducting a full-stack magnetic resonance image segmentation analysis by using the test data set. A subject's full-stack images were given an index number that ranged from 1 to the total number of slices (N). Figure 2 shows a subject's full-stack MRC image patches for the left and right ears and their corresponding segmentation results. The INHEARIT-v2 platform–selected representative slice for each class was indicated by a cyan-colored slice boundary. Figure 2 also shows the segmentation results and the MRC patches for a selected index number and the ground truths of each class. In this example, the selected index number was identical to the ground truths of all classes. The system chose the representative slices for each class—the left cochlea, left vestibule, right cochlea, and right vestibule—based on the size of the segmented area in each class. The representative key slice selection process was formulated as follows:



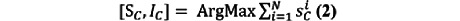



In equation 2, *i* is a slice index, *N* is the total number of segmented images of a subject, *s^i^_c_* represents a segmented area in the *ith* slice in class C, *I_C_* is the key slice index of class C, and S_c_ is the segmented region in the key slice in class C. Thus, the key slices with the largest segmented areas for each class were selected. The slices chosen by the INHEARIT-v2 system and human experts were compared, and the index distance between the slices was calculated. The chosen segmentation results were used as masks for EH ratio calculation in the next step.

The EH ratio was estimated by using a HYDROPS-Mi2 image, which is an image that is generated via the pixel-wise multiplication of HYDROPS and MRC image signals [11] for a given index slice. The EH ratio is defined as follows [12]:


EH ratio = Total number of pixels with a negative value in the segmentation area/total number of pixels in the segmentation area **(3)**

The above equation (equation 3) can be restated as follows:


EH ratio = (*P_C_^Seg^* ∩ *P_C_^Neg^*)/*P_C_^Seg^*** (4)**

In equation 4, *P_C_^Seg^* denotes the total number of pixels in the segmented area in class C, and *P_C_^Neg^* denotes the total number of pixels with negative values in class C.

### Statistical Analysis

The agreement between the values calculated by experts (the neuroradiologist and the neuro-otologist) and the INHEARIT-v2–predicted values was measured by using a single-score intraclass correlation coefficient (ICC), which was based on a two-way model and Pearson correlation coefficient. The analyses were performed by using R software (The R Foundation) [19].

## Results

### Model Experiments

Table 1 shows the segmentation performance of the developed artificial intelligence–based models—the 3into3VGG, 3into3Inception, and 3intoUNet networks. Average performance values were represented as the average IoU values from the fivefold cross-validation with SDs, which were 0.761 (SD 0.036) for 3into3VGG, 0.784 (0.025) for 3into3Inception, and 0.811 (0.032) for 3intoUNet. The U-Net–based model, 3intoUNet, had the highest mean area under the receiver operating characteristic curve. Therefore, the 3intoUNet model was used for the additional test.

**Table 1:** The segmentation performance of the proposed networks—3into3VGG, 3into3Inception, and 3intoUNet. These networks were based on the Visual Geometry Group-19, Inception-v3, and U-Net networks, respectively. The results are represented as the average intersection over union values from the fivefold cross-validation with SDs.

Networks	IoU,^a^ mean (SD)
3into3VGG	0.761 (0.036)
3into3Inception	0.784 (0.025)
3intoUNet	0.811 (0.032)

^a^IoU: intersection over union.

### Additional Test With the Full-Stack Image Data Set

The data set of magnetic resonance images that were collected from 60 new participants (males: n=19; females: n=41; age: mean, 47.1 years, SD 15.27 years; range 21-68 years) for an additional test consisted of 33 subjects with definite Ménière disease (unilateral or bilateral), 17 subjects with sensorineural hearing loss and vertigo, and 10 normal subjects without any symptoms.

System validation was performed on the full-stack images of the subjects. Each image consisted of 104 MRC axial-view image slices, and 3 sequential slices—the main axial-view image slices and the previous and next slices—were fed into the trained model as an input. The model automatically segmented the organs by simultaneously analyzing the three input images and generated a segmentation result as an output. A total of 102 segmented output images were acquired from a subject's stack, and 1 image from each class was selected as a representative result.

**Figure 3:**
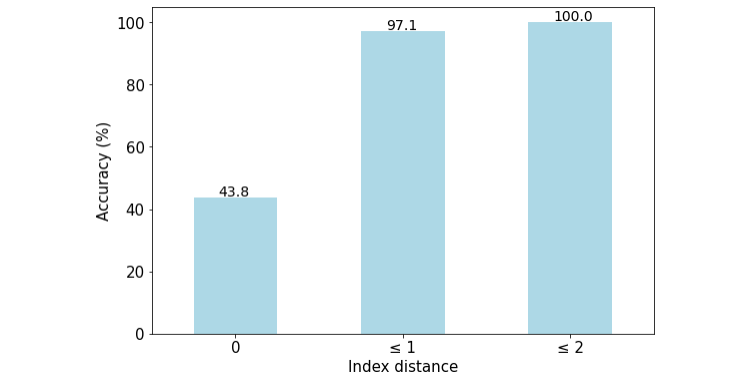
Performance of the inner-ear hydrops estimation via artificial intelligence-version 2 (INHEARIT-v2) system in finding the representative slice index in the magnetic resonance image stacks of 240 target organs of 60 subjects.

With regard to the total 240 image slices of the target organs of 60 subjects, the distance (ie, the number of slice indices) between the manually selected image slices and system-selected image slices was 0 for 105 cases (43.8%), 1 in 233 cases (97.1%), and 2 in 240 cases (100%), as shown by the graph in Figure 3. This means that the representative magnetic resonance slices (ie, from a data set of unseen magnetic resonance images) that were automatically selected by the INHEARIT-v2 system only differed from a maximum of 2 expert-selected slices.

### Agreement Analysis of EH Ratios That Were Calculated by Using the Test Data Set

**Figure 4:**
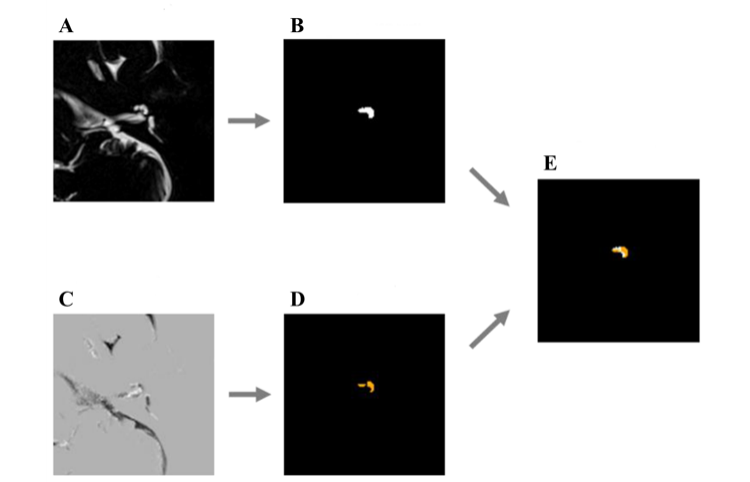
Visualization of an endolymphatic hydrops in a left cochlea. A: Magnetic resonance cisternography patch (left ear). B: Left cochlea segmentation result. C: HYDROPS-Mi2 patch (left ear). D: Endolymphatic hydrops. E: Endolymphatic hydrops in the left cochlea. HYDROPS-Mi2: hybrid of the reversed image of the positive endolymph signal and native image of the positive perilymph signal image multiplied by T_2_-weighted magnetic resonance cisternography.

HYDROPS-Mi2 images were generated at the selected slice index, and EH ratios were calculated for all additional test cases. A case example of segmentation and EH visualization is represented in Figure 4; an MRC patch for a left ear was segmented, and the EH of the left cochlea (as seen in the HYDROPS-Mi2 patch) is shown in orange. The segmented result was used as a mask. A hydrops was overlaid on the segmented result, and the calculated EH ratio was 0.633.

**Figure 5:**
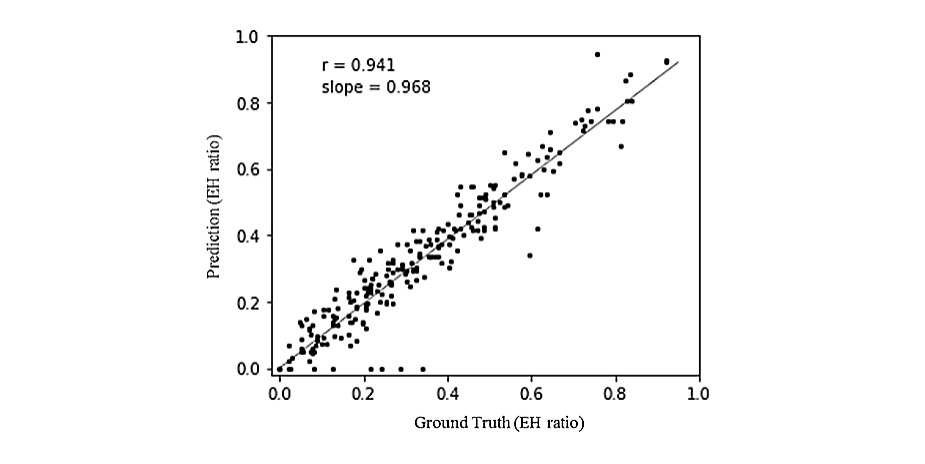
The correlation between physician-calculated (ground truth) EH ratios and those predicted by the proposed platform (based on the segmentation area). EH: endolymphatic hydrops.

The agreement between the physician-calculated and INHEARIT-v2–estimated EH ratios was calculated. The average ICC value for all cases was 0.941; the average ICC of the vestibules was 0.968, and that of cochleae was 0.914 (Figure 5). The average INHEARIT-v2–based calculation time was 3.585 seconds (SD 0.642 seconds) per subject.

## Discussion

### Principal Findings

Due to the development of MRI machines, data and image processing technology, big data, and cloud systems, the role of artificial intelligence in the medical field will likely increase with time. Recently, algorithms have begun to help clinicians in real-world clinics and have been used to predict clinical outcomes that are useful for health care systems [3]. We developed a fully automated analytic system—the INHEARIT-v2 system—for calculating EH ratios by using deep learning and MRI. The INHEARIT-v2 system automatically segmented cochleae and vestibules in brain MRC images and detected representative slices for these organs. The system estimated the EH ratios of segmented regions in key image slices, and the results had a high correlation with those that were manually calculated by experts during an additional test.

Since the mid-2000s, the analysis of EHs by using 3T MRI has been widely accepted as a useful method for the diagnosis of Ménière disease among various groups [10,20]. In particular, injecting contrast media through an intravenous route is a widely used MRI method for patients with Ménière disease because it is less invasive, results in shorter waiting times for patients, and allows physicians to observe both ears simultaneously [11]. Recently, hydropses have been evaluated in several studies either by precisely dividing each area of the cochlea and vestibule or by using a 3D model to measure the whole volume of the endolymphatic space based on magnetic resonance images [21,22].

Recent medical image studies have reported the development of fully automated analytic systems. The implemented tools were designed to provide end-to-end workflows to minimize the amount of human intervention during analysis [23,24]. Several recent studies used deep learning techniques to segment inner ears and related organs in medical images. Some studies were conducted on computed tomography images to segment other organs, such as the sigmoid sinus, facial nerves, or temporal bones [25,26]. Another study analyzed magnetic resonance images for labyrinth segmentation [27]. These studies analyzed various ear-related organs in 3D space, but the main purpose of segmentation was primarily anatomical visualization for surgical planning. However, our study is different because the objective of segmentation was to calculate EH ratios in patients with Ménière disease by conjoining different magnetic resonance image modalities.

The results of our study substantially complemented the limitations of our previous study [12]. Although our former results laid the foundation for conducting EH analyses with deep learning technology, in this study, the improvements allowed for immediate clinical diagnosis and follow-up. Consequently, the accuracy of the EH analysis increased, and even if a human expert did not choose a representative image slice, the representative section was automatically extracted from the magnetic resonance image stack and analyzed successfully.

Another technical improvement over our previous study is that in this study, various deep learning models, such as the U-Net and Inception-v3 networks, were used as a base architecture. We developed a better performing model that effectively segmented inner ears. Furthermore, an additional test was performed to prove the robustness of the system. By developing the INHEARIT-v2 system, expert interpretation became much easier and faster, and more objective analyses were possible.

The best performance, which was based on the mean area under the receiver operating characteristic curve from the fivefold cross-validation, was achieved by the 3intoUNet network, which was based on the U-Net architecture. The U-Net network is designed for medical image segmentation [16]. As such, it is distinguishable from other deep learning models, such as VGG-19 and Inception-v3. Further, it was initially developed for the classification of raw images, such as those of a car, building, or human [13,15]. The 3intoUNet network allows for the analysis of consecutive images, similar to how medical experts browse an MRI stack to identify the location of a target organ. This serial-image training approach was suggested in our previous study on using a VGG-19–based model, which proved to be effective in a medical image segmentation task [12]. The same approach was successful for both the U-Net–based and Inception-v3–based networks. Since inner ears occupy only a small portion of the area (<60 pixels) in whole-brain images (384×324 pixels), the 3intoUNet network has a generalizable architecture for analyzing full-stack magnetic resonance images.

With regard to the performance of the system in automatic representative slice selection, when compared to physicians’ choices, the system’s choices had a gap distance of ≤2 for 100% (240/240) of the test data set. The physicians' goal was to select a key slice of an organ based on the organ's anatomical relationship with other organs [12], whereas the system's goal was to locate the slice with the largest segmented area for each organ. Despite the possible minimal misalignment between the automatically selected and manually selected key slices, the correlation between the INHEARIT-v2–calculated and physician-calculated EH ratio values was high, indicating that the amount of misalignment did not substantially affect the EH ratio calculation. Based on our results, measuring the entire hydrops volume of the cochlea and vestibule for diagnosis is unnecessary.

Several concerns might arise when artificial intelligence systems are used in real clinical settings. In this study, we found that using such artificial intelligence systems could be an alternative to manually measuring hydrops ratios in real settings. However, this does not mean that Ménière disease can only be diagnosed by this system. The cutoff value for the EH ratio was not clearly defined during the diagnosis of definite Ménière disease. Future studies for estimating the EH ratio cutoff value in the diagnosis of definite Ménière disease are still needed. In addition, HYDROPS-Mi2 images were used to analyze EH ratios. However, these images were acquired via software modification, and hydropses in modified images can be more exaggerated compared to those in original images. The final concern is that Ménière disease is a multifactorial disease, which means that making an accurate diagnosis is typically a very complex process. Other diseases that mimic the symptoms of Ménière disease should be ruled out in clinical settings. The main purpose of hydrops measurement via artificial intelligence is to provide clinical support, which can be helpful for medical professionals when making a final clinical diagnosis.

Future studies can include additional normal control subjects to determine the optimal EH ratio threshold by comparing individuals with Ménière disease and healthy individuals without the disease. However, the association between clinical symptoms and EHs is not uniform from patient to patient; thus, such analyses require a clinician's comprehensive judgment [28]. To improve these analyses, in addition to magnetic resonance images, a model for analyzing heterogeneous data, such as the clinical variables used for diagnosis, can be applied to the deep learning algorithm. In addition, because the cause and mechanism of Ménière disease have not been fully elucidated, such technology can be widely used for the differential diagnosis of other conditions that are thought to be associated with EHs [29].

Notably, we fully automated the calculation of EH ratios by developing an analytic system—the INHEARIT-v2 system—by using MRI and deep learning, which have important clinical implications. Although several aspects should be further investigated, this framework will be a helpful tool for clinicians who adopt an MRI analysis approach for diagnosing patients with Ménière disease.

### Conclusion

We developed a fully automated system—the INHEARIT-v2 system—for calculating EH ratios by using deep learning and MRI. The proposed system can quickly and accurately analyze EHs without the intervention of an expert of various inner ear diseases, including Ménière disease experts. This automatic system can perform objective and time-saving analyses for assessing the EH ratios in the inner ear magnetic resonance images of patients with Ménière disease.

## Appendix

Multimedia Appendix 1 The concept of the 3intoUNet network.

## Abbreviations

EH: endolymphatic hydrops
HYDROPS: hybrid of the reversed image of the positive endolymph signal and native image of the positive perilymph signal
HYDROPS-Mi2: hybrid of the reversed image of the positive endolymph signal and native image of the positive perilymph signal image multiplied by T_2_-weighted magnetic resonance cisternography
ICC: intraclass correlation coefficient
INHEARIT: inner-ear hydrops estimation via artificial intelligence
INHEARIT-v2: inner-ear hydrops estimation via artificial intelligence-version 2
IOU: intersection over union
MRC: magnetic resonance cisternography
MRI: magnetic resonance imaging
PEI: positive endolymph image
PPI: positive perilymph image
ROI: region of interest
VGG: Visual Geometry Group
