# Supplementary figures and images for: A Fully Automated Analytic System for Measuring Endolymphatic Hydrops Ratios in Patients With Ménière Disease via Magnetic Resonance Imaging: Deep Learning Model Development Study
Source: J Med Internet Res. 2021 Sep 21;23(9):e29678. doi: 10.2196/29678 (PMC8493456; doi:10.2196/29678)

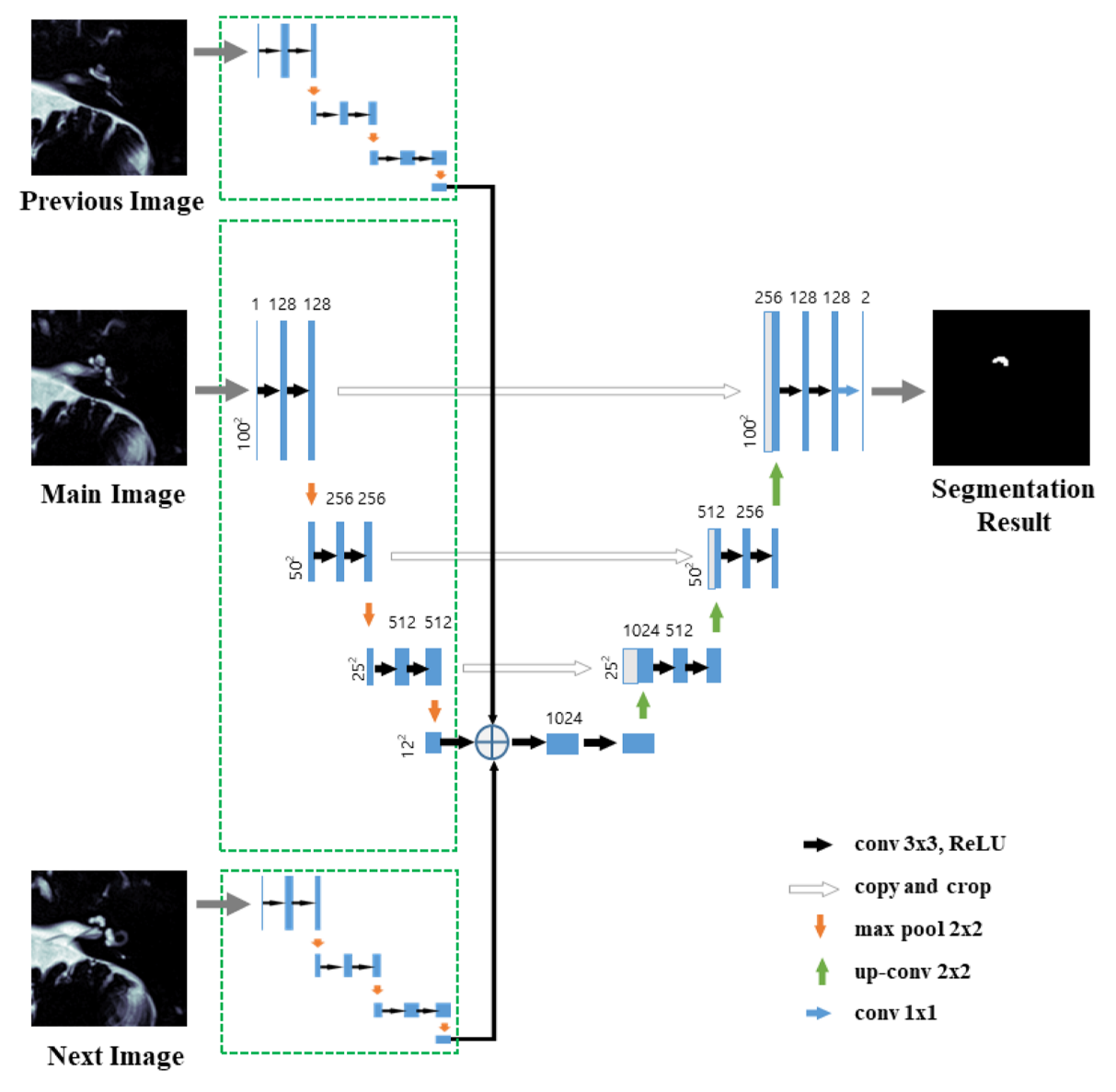

Supplement: Multimedia Appendix 1 [file jmir_v23i9e29678_app1.png]
